# Characterization of lncRNA Profiles of Plasma-Derived Exosomes From Type 1 Diabetes Mellitus

**DOI:** 10.3389/fendo.2022.822221

**Published:** 2022-05-12

**Authors:** Haipeng Pang, Wenqi Fan, Xiajie Shi, Jiaqi Li, Yimeng Wang, Shuoming Luo, Jian Lin, Gan Huang, Xia Li, Zhiguo Xie, Zhiguang Zhou

**Affiliations:** National Clinical Research Center for Metabolic Diseases, Key Laboratory of Diabetes Immunology (Central South University), Ministry of Education, and Department of Metabolism and Endocrinology, The Second Xiangya Hospital of Central South University, Changsha, China

**Keywords:** type 1 diabetes mellitus, exosomes, long non-coding RNA, plasma-derived, bioinformatics analysis

## Abstract

**Backgrounds:**

Exosomes contain several types of transcripts, including long non-coding RNAs (lncRNAs), and have been shown to exert important effects in human diseases. However, the roles of exosomal lncRNAs in type 1 diabetes mellitus (T1DM) have not been well investigated. In the present study, we characterized the plasma-derived exosomal lncRNAs expression profiles of T1DM and predict their potential function in the pathogenesis of T1DM.

**Material and Methods:**

Exosomal lncRNA expression profiles were detected by Illumina Hiseq platform (T1DM subjects N=10; age-, sex- matched Control subjects N=10). Six exosomal lncRNAs were selected to validate their expression level by using quantitative real-time PCR (qRT-PCR) (T1DM subjects N=30; age-, sex- matched Control subjects N=30). Bioinformatics analysis approaches were carried out to explore the potential biological function of differentially expressed lncRNAs.

**Results:**

A total of 162 differentially expressed exosomal lncRNAs were identified in T1DM patients compared with control subjects, among which 77 up-regulated and 85 down-regulated. The expression level of the selected six lncRNAs didn’t show significant difference in the following qRT-PCR analysis. Gene Ontology analysis enriched terms such as activation of phospholipase D activity, neuronal cell body membrane, and calcium sensitive guanylate cyclase activator activity for cis-acting genes of lncRNAs, and metal ion binding for trans-acting genes. The most enriched Kyoto Encyclopedia of Genes and Genomes pathways for the lncRNAs were associated with oxidative phosphorylation and Parkinson’s disease for cis-acting genes, and pathways in cancer as well as focal adhesion for trans-acting genes.

**Conclusions:**

This study characterized the lncRNA profiles of plasma-derived exosomes from T1DM for the first time and these results highlighted the potential role of exosomal lncRNAs in T1DM pathogenesis. A better understanding of exosomal lncRNA profiling will provide novel insights into its molecular mechanisms.

## Introduction

Type 1 diabetes mellitus (T1DM) is a chronic autoimmune disease characterized by absolute insulin deficiency and resultant hyperglycemia (1, 2). The incidence and prevalence of T1DM are increasing worldwide and more than 463 million people are affected (3, 4). This disease is incurable at present and patients with T1DM have to rely on lifelong insulin administration. Besides, the high prevalence of diabetic complications, such as retinopathy, nephropathy, as well as neuropathy, severely influence the life quality of T1DM patients and impose a considerable financial burden. The pathogenesis of T1DM is extremely complex and multiple factors, including genetic backgrounds, environmental triggers, and behavioral changes, may contribute to the onset and development of T1DM (5–7). An improved understanding of T1DM pathophysiology will render early identification and intervene of T1DM.

Growing evidence has suggested that a small extracellular vesicle (EV), namely exosome, plays an important role in multiple pathogenic processes, including T1DM (8). Exosomes, which can be released by virtually all cell types, are significant mediators of intercellular communication and interorgan crosstalk through transferring bioactive molecules, including proteins, lipids, and RNAs between cells (9, 10). Moreover, the content of exosomes is strictly regulated in response to all kinds of endogenous or exogenous stimulations, thus reflecting the biological events or disease conditions (11). Therefore, a better understanding of exosomes may provide a valuable target for disease diagnosis and treatment.

Long non-coding RNAs (lncRNAs), a sub-class of non-coding RNA family, have emerged as crucial regulators in multiple pathophysiological conditions (12). LncRNAs are more than 200 nucleotides in length and take part in the regulation of various cellular and biological processes, such as gene silencing, histone modifications and DNA methylation (13, 14). Accumulating evidence has highlighted the role of lncRNAs in pancreatic islets and development of T1DM (15–18). However, the research about the exosomal lncRNA is relatively lacking, especially in T1DM. Here, we investigated the plasma-derived exosomal lncRNA expression profiles in patients with T1DM and explored their biological function. This present work provided a novel insight into the pathogenesis of T1DM and laid a foundation for using exosomal lncRNAs as biomarkers and therapeutic targets for T1DM.

## Materials and Methods

### Study Subjects

Patients with T1DM attending the Diabetes Clinic at the Second Xiangya Hospital were recruited. The inclusion criteria of case group were as follows: (1) fulfilling the WHO diagnostic criteria for diabetes (1999); (2) acute onset and insulin dependency within 6 months after diagnosis; (3) positive for at least one following islet autoantibodies: GADA (glutamic acid decarboxylase antibody), IA-2A (protein tyrosine phosphatase antibody), or ZnTA8 (zinc transporter 8 antibody); (4) diabetes duration less than 5 years. Exclusion criteria included pregnancy, combined with other autoimmune diseases, malignant tumors, or a recent cardiovascular event. Non-diabetic subjects without autoimmune diseases, cancers, or family history of diabetes were recruited as health control. This case-control study was approved by the Ethics Committee of the Second Xiangya Hospital and all the research methods were conducted in accordance with the ethical guidelines of the Declaration of Helsinki. All the participants or their guardians indicated that they fully understand the research goals and procedures, and written informed consent was obtained. A total of 10 cases and 10 controls were included in the discovery phase. There was no significant difference in age (*P*=0.97) and sex (*P*=0.36) between two groups (**Table 1**). Besides, we used a new cohort (T1DM subjects N=30; age-, sex- matched Control subjects N=30) for the following qRT-PCR analysis.

**Table 1:** Characteristics of T1DM and control subjects.

Characteristic	T1DM (n=10)	Control (n=10)	*P* value
Sex (male/female)	3/7	5/5	0.36
Age (year)	25.20 ± 7.24	25.10 ± 2.96	0.97
BMI (kg/m2)	21.26 ± 2.71	20.40 ± 2.24	0.47
Duration (months)	25.40 ± 14.94	–	–
FPG (mmol/L)	7.79 ± 3.32	–	–
HbA1c %	8.01 ± 2.20	–	–

BMI, body mass index; FPG, fasting plasma glucose; HbA1c, Hemoglobin A1c.

### Isolation of Plasma

Peripheral blood was collected in the EDTA blood tubes from each participant and plasma was separated by centrifugation at 3000 g for 15 min at 4°C. The plasm samples were stored at -80°C before use.

### Isolation of Exosome

Size exclusion chromatography methods were adopted to isolate exosomes. In brief, 1 mL of 0.8 μm filtered plasma was 1.5-fold diluted with phosphate-buffered saline (PBS) and the further purification was made by using Exosupur^®^ columns (Echobiotech, China). Next, we eluted the samples with 0.1M PBS and collected about 2 mL elute fractions. The collected fractions were then concentrated to 200 μL by 100 kDa molecular weight cut-off Amicon^®^ Ultra spin filters (Merck, Germany). All procedures were operated according to the manufacture’s institutions.

### Transmission Electron Microscopy (TEM)

We put 10 µL purified exosome on a copper mesh and the sample was incubated at room temperature for 1 min. The exosome was negatively stained with uranyl acetate solution for 1 min after washing with sterile distilled water. After dried for 2 min under incandescent light, the exosome was examined under a TEM (H-7650, Hitachi Ltd., Tokyo, Japan).

### Nanoparticle Tracking Analysis (NTA)

The size distribution and quality of isolated particle (concentrations between 1x10^7^/mL and 1x10^9^/mL) were determined by the ZetaView PMX 110 (Particle Metrix, Meerbusch, Germany), which equipped with 405 nm laser. A 60 second video was taken with a frame rate of 30 frames/second. The, the particle movement was analyzed by NTA software (ZetaView 8.02.28).

### Western Blot Analysis (WB)

The exosome supernatant was denatured in 5 × sodium dodecyl sulfonate (SDS) buffer and subjected to WB analysis (10% SDS-polyacrylamide gel electrophoresis; 50 µg protein/lane) using rabbit polyclonal antibody CD63, TSG101, Alix and calnexin. The proteins were visualized on the Tanon4600 Automatic chemiluminescence image analysis system (Tanon, Shanghai, China). The identification of exosome, including TEM, NTA, and WB analysis, was entrusted the company (Echo Biotech Co., Ltd, Beijing, P. R. China).

### Total RNA Isolation

The miRNeasy Serum/Plasma Advanced Kit (Qiagen, cat. No. 217204) was used to extract and purify the exosomal RNA in compliance with the kit instruction. The concentration and purity of RNA were evaluated using the RNA Nano 6000 Assay Kit of the Agilent Bioanalyzer 2100 System (Agilent Technologies, CA, USA).

### Library Preparation and Sequencing

The extracted RNA was used as input material for sequencing libraries using the SMARTer Stranded Total RNA-Seq Kit (Takara Bio USA, Inc.) according to manufacturer’s recommendations. Index codes were added to attribute sequences to each sample. At last, library quality was evaluated on the Agilent Bioanalyzer 2100 and qPCR. The clustering of the index-coded samples was performed on acBot Cluster Generation System using TruSeq PE Cluster Kitv3-cBot-HS (Illumina, San Diego, CA, USA). After cluster generation, the library preparations were sequenced on an Illumina Hiseq platform and paired-end reads were generated.

### LncRNA Analysis

The transcriptome was assembled using the Stringtie and Scripture based on the reads mapped to the reference genome. The assembled transcripts were annotated using the Cuffcompare program from the Cufflinks package. The unknown transcripts were used to screen for putative lncRNAs. Four computational approaches including CPC, CNCI, Pfam, and CPAT were combined to elect ncRNA candidates from presumed protein-coding RNAs in the unknown transcripts. Presumed protein-coding RNAs were filtered out using exon number threshold and a minimum length. Transcripts having more than two exons and with lengths more than 200 nt were selected as lncRNA candidates and further screened using CPC, CNCI, Pfam, and CPAT that have the power to distinguish the protein-coding genes from the non-coding genes. As well as the different types of lncRNAs include long intergenic lncRNA (lincRNA), intronic lncRNA, anti-sense lncRNA were selected using cuff compare. Stringtie was used to calculate FPKMs of lncRNAs in each sample. Gene FPKMs (fragments per kilo-base of exon per million fragments) were computed by summing the FPKMs of transcripts in each gene group. The lncRNA sequencing and lncRNA analysis were entrusted the company (Echo Biotech Co., Ltd, Beijing, P. R. China).

### GO and KEGG Pathway Enrichment Analysis

Gene Ontology (GO) enrichment analysis of the differentially expressed lncRNAs was implemented by the topGO R packages. We used KOBAS (19) software to test the statistical enrichment of differential expression genes in Kyoto Encyclopedia of Genes and Genomes (KEGG) pathways (http://www.genome.jp/kegg/).

### Quantitative Real-Time PCR (qRT-PCR) Assay

The total RNA from exosomes was extracted using miRNeasy Serum/Plasma Advanced Kit (Qiagen, cat. No. 217204) according to the manufacturer’s protocol. The total RNA was then reverse transcribed to synthesize cDNA using PrimeScript™ RT reagent Kit (Perfect Real Time) (TAKARA, RR037A). The abundance of target gene expression was detected by TaqMan^®^ probe using qRT-PCR. 2 µL of cDNA was used as the template for each PCR reaction. The GADPH was selected as the internal reference gene. The sequence of primers and probes were shown as **Table 2**.

**Table 2:** Primer list. F- refers to the forward sequence, R- refers to the reverse sequence, and P- refers to the probe sequence.

LncRNA	Symbol	Primer Sequence (5’-3’)
ENST00000533796	TALDO1-209	F-GATGCTACCACCAACCCG
		R-AATTCGGTCCTTGCTGATCC
		P-CCGGAAGCTGGGCGGGTCAC
ENST00000472614	GNB1-207	F-GAGGGCGCTGAGACAAATT
		R-GTGCTCTTCAATGCCACCTT
		P-CCAGACCAGAAGCCCTTCTGAATTAAG
ENST00000525207	SF3B2-204	F-GCTGAAGGAGAGCCGC
		R-TGTCCTCCTCAGTCTCTGAC
		P-AGGAAATGGAAACAGATGCTCGCT
ENST00000495420	ANP32A-208	F-GAGCCTTCAAAGTCCTAAAACG
		R-TTCATTCGACCGACTGTTGT
		P-AGAGCTGCGGAACAGGACG
ENST00000488260	EIF1B-204	F-CGGCAGGGACTGAGGATT
		R-ATCACAGTACCATTACAGGCAAA
		P-ATCCAGCAACGGAACGGCA
ENST00000505090	CANX-210	F-TTTCAGCCAGGCGTTGTG
		R-CTCCTCATCTCCCTTGTCCT
		P-CCCGTGGCTGTGGGTAGTC

### Statistical Analysis

We used SPSS software version 20.0 to perform the statistical analysis. The data were presented as mean ± SD (standard deviation). Differential expression analysis of lncRNAs between two groups was performed using the Mann Whitney U test with cutoff FPKM > 5, *P*-value < 0.05 and Fold change > 1.5. The comparisons of the relative expression level of candidate lncRNAs in qRT-PCR were analyzed by the unpaired t-test. A *P*-value less than 0.05 was considered as statistically significant.

## Results

### Characterization of Exosome

**Figure 1:**
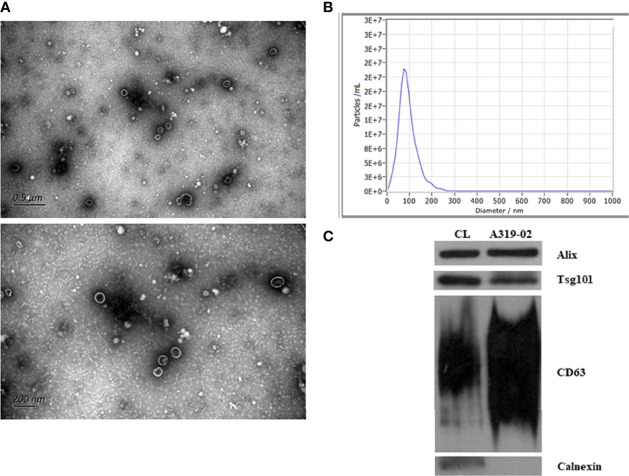
Identification of exosomes. The transmission electron microscopy images of exosomes **(A)**. The size distribution of exosomes analyzed by nanoparticle tracking analysis **(B)**. Validation of marker proteins and negative protein of exosomes **(C)**.

We used TEM and NTA to evaluate the morphology and sizes distribution of isolated exosome. As shown by **Figure 1**, the isolated particles were oval and cup-shaped with diameter range between 30 nm to 200 nm (median, 91.7 nm). WB analysis indicated that the presence of exosomal positive protein markers Alix, Tsg101, and CD63, and the absence of Calnexin, the negative protein marker for exosome. All these results suggested that the isolated exosomes were well-prepared and with good purity.

### An Overview of the RNA Sequencing Results

**Figure 2:**
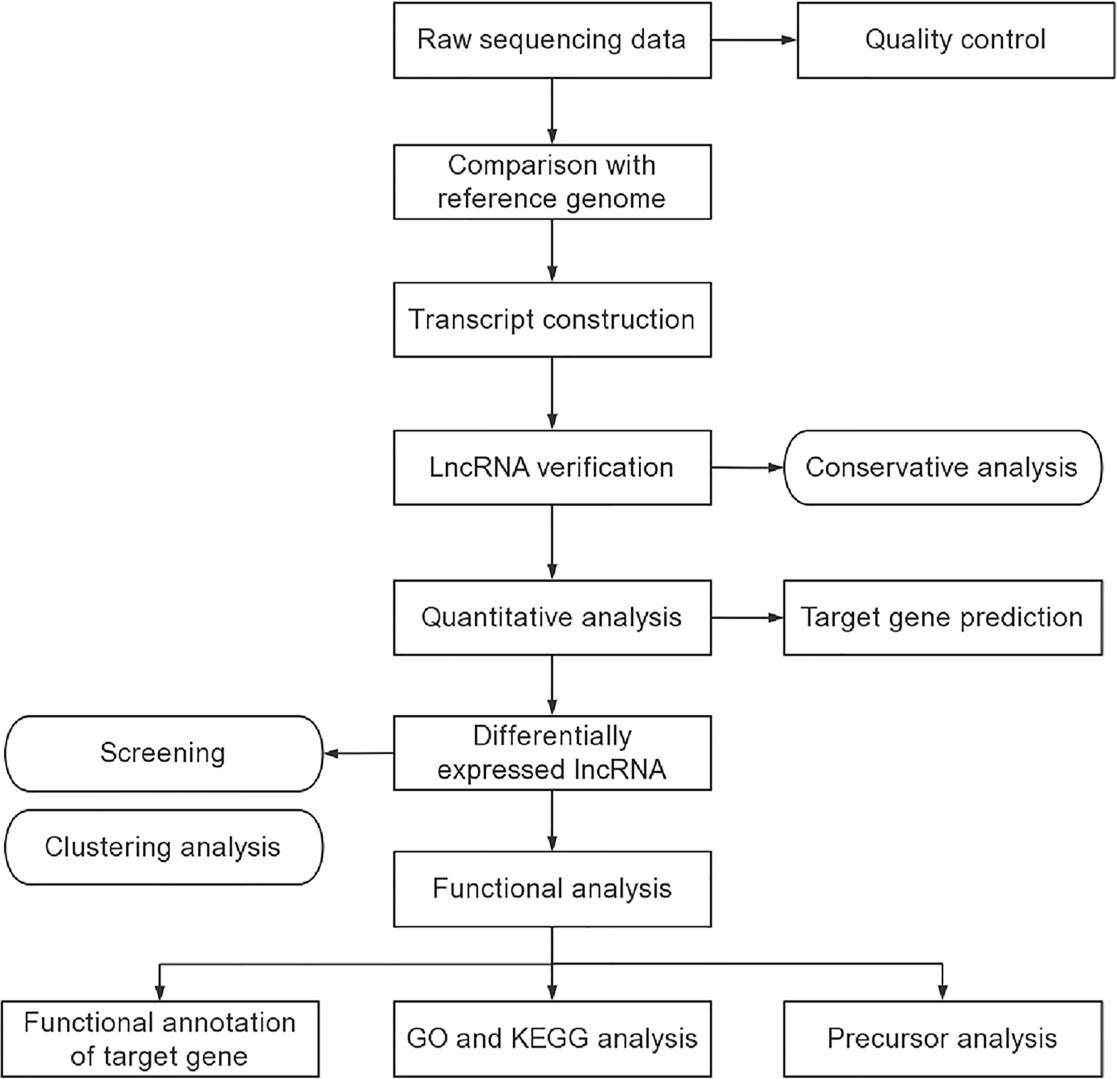
The procedures of exosomal lncRNA sequencing and bioinformatics analysis.

The procedures of lncRNA sequencing and bioinformatics analysis were summarized in **Figure 2**. A total of 20 samples, including 10 patients and 10 healthy controls were constructed and analyzed. The percentage of Q30 base was at least 91.94%. A total of 40065 lncRNAs, including 33453 known lncRNAs and 6612 novel lncRNAs were generated. Among these identified lncRNAs, lincRNAs accounted for the largest proportion (69.1%), followed by intronic lncRNAs (15.5%), sense lncRNAs (8.6%), and antisense lncRNAs (6.8%). Then, we predicted the target genes of lncRNA. On the one hand, it is predicted that the adjacent genes within 100 kb were its cis-acting gene because lncRNA could regulate the expression of neighboring genes. On the other hand, the trans-acting genes of the lncRNA were predicted by analyzing the expression correlation between lncRNA and corresponding mRNA. The genes with absolute Pearson correlation coefficient greater than 0.9 and *P*-value less than 0.01 were taken as the trans-acting genes of the lncRNA.

### Differentially Expressed Exosomal lncRNAs

**Figure 3:**
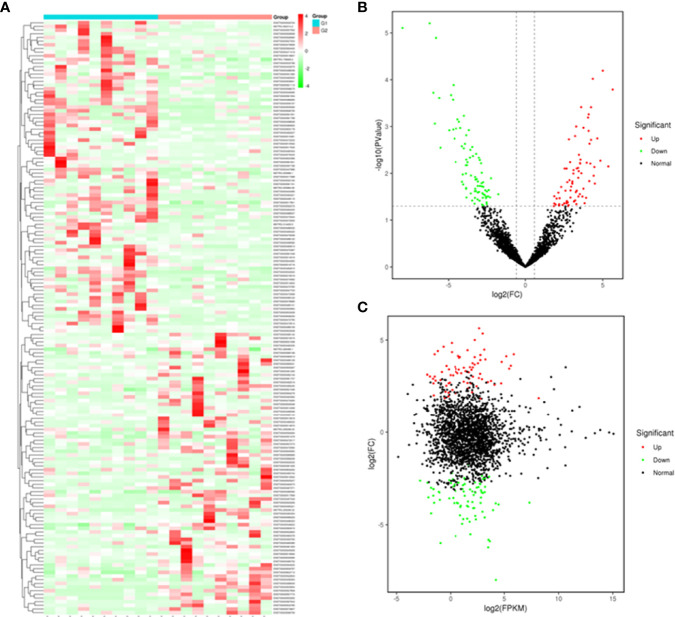
Plasma-derived exosomal lncRNA profiles of case and control group. A heatmap **(A)**, volcano diagram **(B)**, and MA plot **(C)** of the 162 differentially expressed exosomal lncRNAs in T1DM and control subjects. G1: control group; G2: case group.

Based on the plasma-derived exosomal lncRNA expression profiles, differentially expressed lncRNAs were distinguished between T1DM patients and control subjects. Hierarchical clustering analysis was performed to cluster lncRNAs according to their expression level (**Figure 3A**). As shown by volcano diagram (**Figure 3B**) and MA plot (**Figure 3C**), total of 162 differentially expressed lncRNAs were identified, in which 77 up-regulated and 85 down-regulated (**Table 3**). These results indicated that plasma-derived exosomal lncRNA expression profiles were distinguishable between T1DM patients and control subjects.

**Table 3:** The detailed information of the differentially expressed exosomal lncRNAs.

ID	Symbol	*P*-value	log2FC	Regulated
ENST00000533796	TALDO1-209	6.24E-06	-6.22261	down
ENST00000547703	MYL6-208	7.84E-06	-7.97936	down
ENST00000472614	GNB1-207	1.28E-05	-5.81213	down
ENST00000525207	SF3B2-204	6.42E-05	5.004214	up
ENST00000514674	CCT5-212	9.56E-05	4.352669	up
ENST00000514918	NSA2-203	0.00013006	-4.66882	down
ENST00000515015	IK-208	0.00016215	5.635708	up
ENST00000436115	NDUFS7-204	0.000189895	-5.98882	down
ENST00000496582	RPL35A-208	0.000212962	-4.83713	down
ENST00000620389	SRSF3-207	0.000245529	-5.60859	down
ENST00000495420	ANP32A-208	0.000265961	-4.70741	down
ENST00000585223	DDX5-233	0.000385727	3.60407	up
ENST00000587642	WBP2-207	0.000387307	4.236054	up
ENST00000565031	NDUFB10-203	0.00054778	4.086463	up
ENST00000472568	ADD3-207	0.000589455	-3.97259	down
ENST00000531008	RPS13-207	0.000641612	4.050101	up
ENST00000573505	ALDH3A2-210	0.000700788	-4.38782	down
ENST00000550184	MYL6-216	0.00078891	-4.29868	down
ENST00000484600	ARPC1B-218	0.000864268	-5.89173	down
ENST00000466129	RPS24-207	0.000999187	3.280526	up
ENST00000492519	PABPC4-217	0.001059294	3.753608	up
ENST00000488260	EIF1B-204	0.001073793	-4.21143	down
ENST00000561048	PPIB-203	0.001105467	-4.49263	down
ENST00000489109	ANXA1-206	0.001150385	-4.69253	down
ENST00000502548	RACK1-202	0.00119424	-3.65467	down
ENST00000526990	EHBP1L1-202	0.001204172	-4.95234	down
ENST00000472560	AP2M1-216	0.001238868	4.079642	up
ENST00000498038	HLA-DPB1-217	0.001533537	-3.81907	down
ENST00000645908	RPL5-210	0.001592047	3.212761	up
ENST00000496323	SPTBN1-205	0.001789333	4.641172	up
ENST00000469120	HLA-DPB1-213	0.001805199	-4.60675	down
ENST00000525256	C11orf58-206	0.001879295	2.814152	up
ENST00000465744	RPL36A-205	0.001922473	4.237297	up
ENST00000486002	ATP5PF-208	0.002327309	4.141541	up
ENST00000505090	CANX-210	0.002345547	-3.90611	down
ENST00000556167	PSMA6-212	0.002637017	-4.10496	down
ENST00000515592	IK-209	0.002727177	-3.42825	down
ENST00000470287	FUBP1-205	0.00283803	-5.51764	down
ENST00000462527	EIF3A-202	0.002920393	-3.97697	down
ENST00000532520	CASP1-214	0.003146632	-3.55369	down
ENST00000526908	RPS2-204	0.003829729	-3.1769	down
ENST00000585199	RPL19-206	0.004011114	3.457337	up
ENST00000518716	PABPC1-207	0.004281746	-3.39323	down
ENST00000578430	SRSF1-202	0.004590845	-3.57807	down
ENST00000485101	EIF4A2-219	0.004613042	-3.00297	down
ENST00000466142	PPIG-209	0.005074734	3.555666	up
MSTRG.562314.2	MSTRG.562314.2	0.005080913	-3.81647	down
ENST00000508901	BTF3-204	0.005170977	-2.94875	down
ENST00000529348	ZBTB44-208	0.005368448	4.84602	up
ENST00000472755	TWF2-202	0.005711529	-4.52274	down
ENST00000594525	NOP53-203	0.005764486	2.889701	up
ENST00000486032	COPS3-208	0.005778239	-3.74238	down
ENST00000522843	SDCBP-210	0.00593818	3.958216	up
ENST00000466598	AP2M1-214	0.006030961	3.528153	up
ENST00000417088	RPS28-201	0.006071702	-2.86162	down
ENST00000645950	CD99-213	0.006158913	4.194786	up
ENST00000474260	SLK-203	0.00616617	3.539578	up
ENST00000498648	STRADB-210	0.00666578	-3.77403	down
ENST00000581741	RPL17-213	0.006706059	-3.84491	down
ENST00000557774	NUMB-226	0.007099271	5.353631	up
ENST00000493793	EPS15-209	0.007329302	4.457347	up
ENST00000494573	LDHA-211	0.007632476	3.402206	up
ENST00000598706	RAB11B-203	0.007829597	3.324837	up
ENST00000525232	RPL8-203	0.0078692	2.535549	up
ENST00000521046	PCMTD1-205	0.007879014	2.663607	up
ENST00000530585	RPL27A-207	0.008506999	3.108377	up
ENST00000368545	TPM3-213	0.008952212	3.442714	up
ENST00000514842	RPL9-210	0.009683951	-2.82285	down
ENST00000489392	RPL7A-207	0.009940847	3.616313	up
ENST00000598273	SELENOW-206	0.010074218	-3.84098	down
ENST00000460085	ATP6V1E1-205	0.010093959	3.098422	up
ENST00000464240	LAMTOR5-202	0.010535877	-4.04501	down
ENST00000517545	PCM1-202	0.011110826	-3.51861	down
ENST00000612642	AMD1-207	0.011229415	4.406694	up
ENST00000471419	NONO-209	0.011969858	-3.24716	down
ENST00000565143	PKM-214	0.012431727	3.411681	up
ENST00000488122	RPLP1-204	0.012715911	-2.21776	down
ENST00000561798	UQCRC2-203	0.013241519	-3.13868	down
ENST00000648735	HBG1-203	0.013384842	-3.20313	down
ENST00000557552	AKT1-218	0.013650708	-3.22111	down
ENST00000489732	NAMPT-212	0.013977634	2.846161	up
MSTRG.255299.33	MSTRG.255299.33	0.01460306	4.127691	up
ENST00000591551	ZNF224-208	0.014911883	-3.06762	down
ENST00000513816	RAPGEF2-212	0.014928853	3.037927	up
ENST00000600178	ECH1-212	0.01513054	-4.09493	down
ENST00000460454	TRIM22-209	0.01542483	2.704678	up
ENST00000564440	PKM-212	0.015477651	-2.72849	down
ENST00000555863	NEMF-207	0.016201769	-2.83837	down
ENST00000492144	EIF4A2-222	0.016368381	3.027909	up
ENST00000505854	SNX2-202	0.016442341	4.485358	up
ENST00000460378	EPB41-208	0.016492727	3.034939	up
ENST00000503439	ANAPC13-202	0.017240099	-4.01868	down
ENST00000527304	MAP3K11-206	0.018546951	-3.0729	down
ENST00000475808	GLUL-208	0.018551696	-2.92786	down
ENST00000432675	EIF3D-205	0.018957454	-2.80598	down
ENST00000436354	UBXN1-203	0.019178124	2.954689	up
ENST00000521112	RPL30-208	0.019372092	-2.7188	down
ENST00000564276	PKM-211	0.019467707	2.308757	up
ENST00000518657	NDUFB9-205	0.020492678	-3.82501	down
ENST00000490221	CCT3-214	0.021255056	3.750684	up
ENST00000490613	PSMB8-205	0.021316098	-3.24049	down
ENST00000581631	SMCHD1-206	0.021359933	-3.83403	down
ENST00000484615	RPL3-217	0.022749903	-2.36799	down
ENST00000561554	RPL4-202	0.023050097	-2.59119	down
ENST00000561707	COTL1-202	0.023163569	2.919411	up
ENST00000573807	GPS2-213	0.023431442	3.318582	up
ENST00000583515	RPL26-207	0.02348098	2.5861	up
ENST00000646230	PTPRC-217	0.023923166	-3.19677	down
ENST00000578995	SMARCE1-215	0.02397642	-3.26826	down
ENST00000529724	PPP1CA-206	0.02428061	-2.47998	down
ENST00000470944	SOD1-203	0.02495092	-2.99316	down
ENST00000472217	USF1-204	0.025114915	3.260114	up
MSTRG.314430.5	MSTRG.314430.5	0.025574578	-3.07342	down
ENST00000481353	PDCD4-206	0.025896515	2.12976	up
ENST00000531282	NDUFS8-213	0.026490099	-2.77951	down
ENST00000472202	ATP5F1C-207	0.026862338	-3.52936	down
ENST00000527808	BIRC2-203	0.027777176	3.512334	up
MSTRG.739926.2	MSTRG.739926.2	0.02792067	-1.75089	down
ENST00000488537	TXNIP-203	0.02824563	-2.7104	down
ENST00000515481	HNRNPH1-227	0.029065048	-2.62042	down
MSTRG.926466.1	MSTRG.926466.1	0.03085915	1.863832	up
ENST00000517801	CYRIB-204	0.031092289	-2.41059	down
ENST00000534765	SF3B2-216	0.031187865	3.422181	up
ENST00000553252	SLC38A2-213	0.03125579	3.678198	up
MSTRG.505966.1	MSTRG.505966.1	0.031515408	-2.5263	down
ENST00000480304	YWHAZ-212	0.032040453	-2.55559	down
ENST00000474582	RPS8-205	0.032559983	-2.28222	down
MSTRG.505964.56	MSTRG.505964.56	0.032725433	-3.80789	down
ENST00000461935	SRSF11-206	0.032876738	2.915137	up
ENST00000497196	DDX17-210	0.033142368	-2.75023	down
ENST00000467349	MRPL53-203	0.033214202	3.922429	up
ENST00000484221	SLAMF7-208	0.033344736	-3.25879	down
ENST00000644260	DDX3X-240	0.033743268	-2.55081	down
ENST00000519594	LCP2-203	0.033881721	2.208504	up
ENST00000491294	ARPC1B-219	0.034432197	2.016897	up
ENST00000479338	EIF4H-203	0.036070802	-3.09132	down
ENST00000467371	SPTBN1-204	0.037587984	3.216338	up
ENST00000216019	DDX17-201	0.03788691	-3.43456	down
ENST00000447986	ERGIC3-209	0.038357687	-2.96389	down
ENST00000589236	WBP2-209	0.038993347	2.685374	up
ENST00000490335	RPL10A-205	0.03906529	3.182939	up
ENST00000485245	TMEM131-208	0.039873678	3.134736	up
ENST00000484513	MYD88-208	0.040371938	2.666172	up
ENST00000434385	STK38L-203	0.041597845	-2.50685	down
ENST00000602712	MARCHF8-207	0.041761876	2.7343	up
ENST00000517569	SARAF-202	0.042059641	2.656393	up
MSTRG.255299.32	MSTRG.255299.32	0.043398782	3.823257	up
ENST00000612499	IFI27-205	0.043600733	2.662225	up
ENST00000551678	MARF1-210	0.04372356	2.509668	up
ENST00000509797	RUFY1-213	0.043942792	1.86348	up
ENST00000592372	SLC25A39-215	0.044425736	-3.28037	down
ENST00000585695	SLC25A39-206	0.045292114	3.294733	up
ENST00000472760	PRPF40A-208	0.045362214	-2.4923	down
ENST00000507273	RACK1-216	0.045566475	2.184397	up
ENST00000555267	CALM1-211	0.046144992	1.959441	up
ENST00000548485	OSBPL8-208	0.046195315	-2.79872	down
ENST00000522823	LCP2-208	0.04718699	2.405215	up
ENST00000602252	RPS11-209	0.047579066	2.795051	up
ENST00000594264	USF2-206	0.048591173	2.165011	up
ENST00000460633	HLA-DQA1-205	0.048849575	-2.73019	down
ENST00000493362	HNRNPK-211	0.049736378	2.755326	up
ENST00000548622	HSP90B1-204	0.049899032	1.839573	up

### Functional Analysis of Exosomal lncRNAs

**Figure 4:**
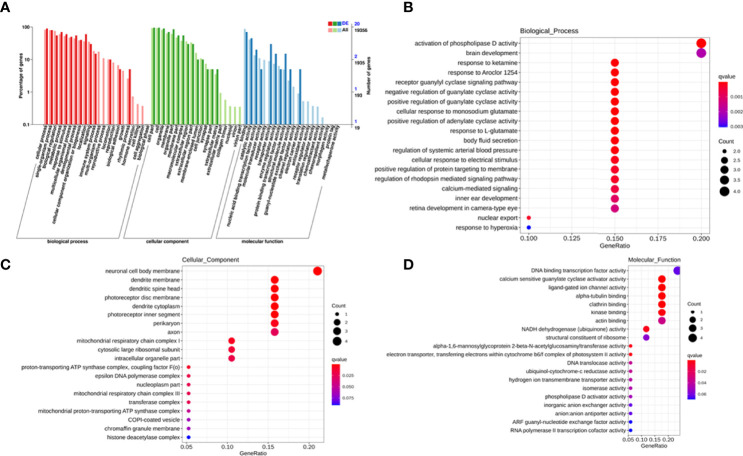
GO terms of cis-acting genes of differentially expressed exosomal lncRNAs. GO categories of differentially expressed lncRNAs **(A)**. Top 20 significant enriched biological processes **(B)**, cellular components **(C)**, and molecular functions **(D)** of differentially expressed lncRNAs.

**Figure 5:**
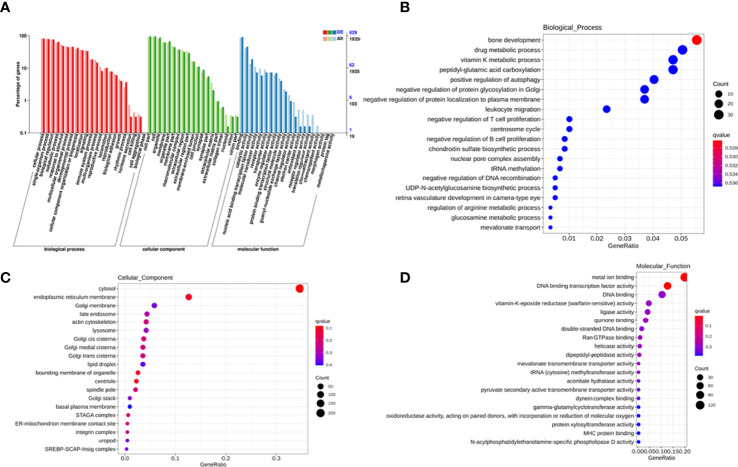
GO terms of trans-acting genes of differentially expressed exosomal lncRNAs. GO categories of differentially expressed lncRNAs **(A)**. Top 20 significant enriched biological processes **(B)**, cellular components **(C)**, and molecular functions **(D)** of differentially expressed lncRNAs.

**Figure 6:**
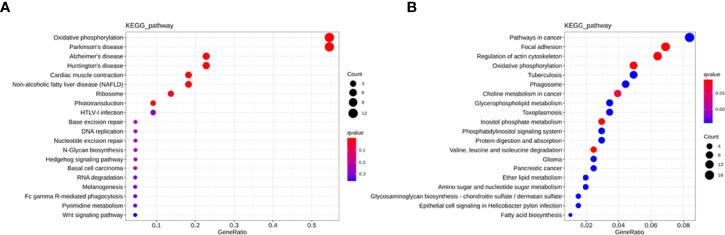
KEGG pathway analysis for cis-acting genes **(A)** and trans-acting genes **(B)** of differentially expressed lncRNAs in T1DM and control subjects.

To explore the potential biological function of 162 differentially expressed lncRNAs, GO enrichment analysis and the KEGG pathway analysis were employed. The lncRNAs exert biological effects by acting as cis- or trans- regulators (20, 21). We summarized the significantly enriched GO terms of cis-acting genes of differentially expressed lncRNAs regarding to the biological process (BP), cellular component (CC), and molecular function (MF), respectively (**Figure 4A**). For BP, the term with the most genes and with the highest enrichment score was the activation of phospholipase D activity (GO:0031584) (**Figure 4B**). For CC, the term with the most genes was the extracellular exosome (GO:0070062) and with the highest score was neuronal cell body membrane (GO:0032809) (**Figure 4C**). In addition, for MF the terms DNA binding transcription factor activity (GO:0003700) and protein binding (GO:0005515) ware associated with most genes, and the most enriched term was calcium sensitive guanylate cyclase activator activity (GO:0008048) (**Figure 4D**). We also summarized the GO terms of trans-acting genes (**Figure 5A**). For BP, the term with the most genes was the positive regulation of transcription from RNA polymerase II promoter (GO:0045944) (**Figure 5B**). For CC, the term cytosol (GO:0005829) was correlated to most genes (**Figure 5C**). For MF, the term with protein binding (GO:0005515) was most genes, and metal ion binding (GO:0046872) was most significantly enriched term (**Figure 5D**). Besides, the enriched pathways of target genes of lncRNA were analyzed by KEGG. For cis-acting genes, the most significantly enriched terms were involved in oxidative phosphorylation (ko00190) and Parkinson’s disease (ko05012) (**Figure 6A**). For trans-acting genes, the terms pathways in cancer (ko05200) and focal adhesion (ko04510) were related to most genes (**Figure 6B**). We also performed the disease annotation of identified lncRNAs through sequence alignment with LncRNADisease database. The results showed some lncRNAs were associated with autoimmune diseases, including dermatomyositis and polymyositis. LncRNA can be precursor molecule of miRNA. We also detected the lncRNAs which serve as precursor of miRNA through comparing and predicting the miRNA sequence in miRbase database.

### Validation of Exosomal lncRNA Expression by qRT-PCR

**Figure 7:**
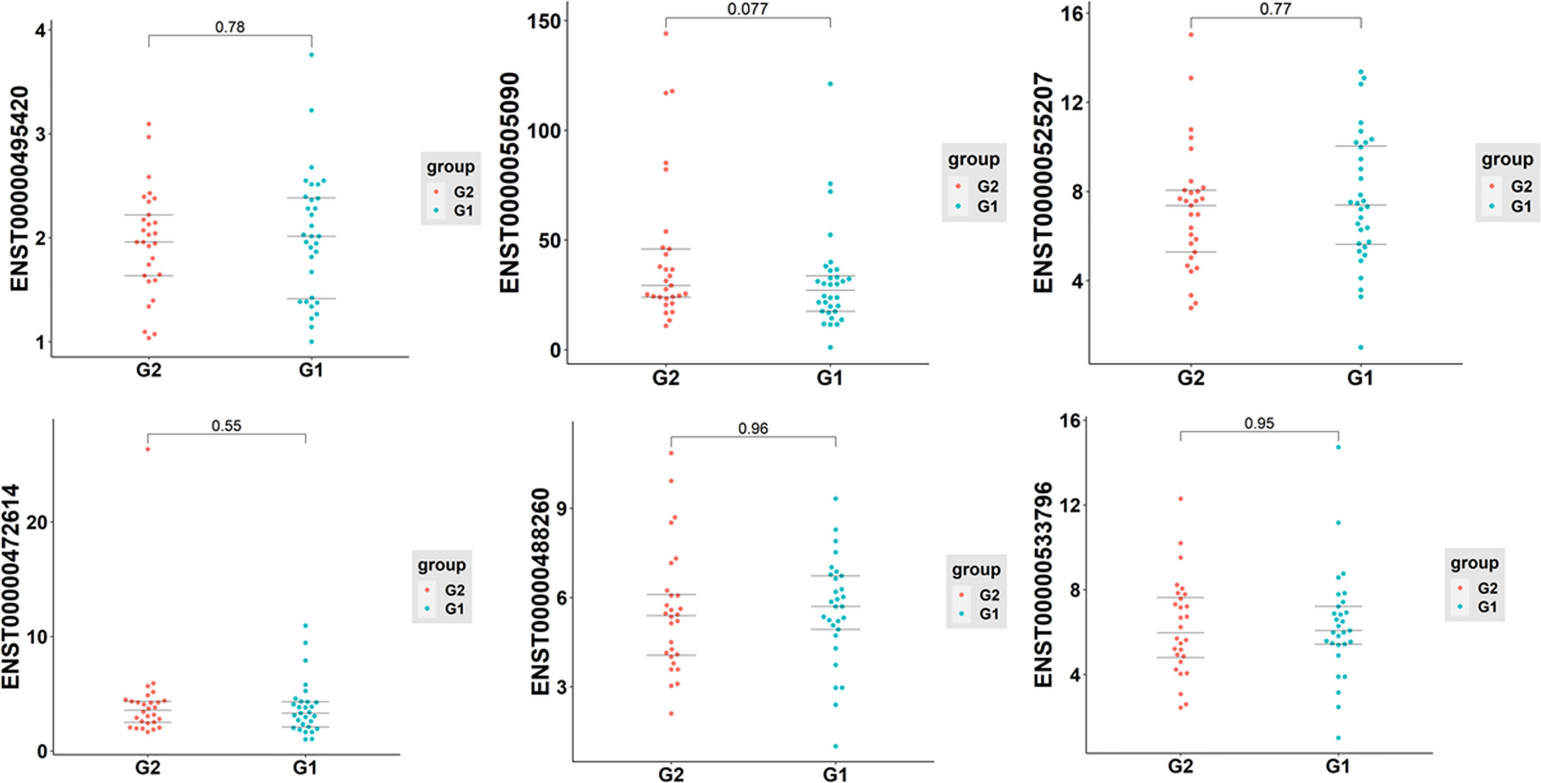
The qRT-PCR analysis of selected six exosomal lncRNAs in T1DM and control subjects (T1DM subjects N=30; age-, sex- matched Control subjects N=30). G1: control group; G2: case group.

To further verify the sequencing data, six differentially expressed exosomal lncRNAs, including one up-regulated (ENST00000525207) and five down-regulated (ENST00000495420, ENST00000488260, ENST00000505090, ENST00000472614, and ENST00000533796), were randomly selected to perform the qRT-PCR analysis (T1DM subjects N=30; age-, sex- matched Control subjects N=30). However, the results indicated that the expression levels of selected lncRNAs were similar between T1DM patients and control individuals (**Figure 7**).

## Discussion

Nowadays, it has been reached a consensus that T1DM is a multifactorial disease, and the precise pathogenic mechanisms are still obscure. Mounting evidence has suggested exosomes play an important role in the onset and development of T1DM (8, 22). However, research about the expression profiles of exosomal lncRNA in T1DM has not yet been reported. Here, we performed the transcriptome-wide expression patterns of plasma-derived exosomal lncRNAs in the T1DM using Illumina Hiseq platform, and further explore their function by bioinformatics analysis.

In the present study, 162 aberrantly expressed exosomal lncRNAs, including 77 up-regulated and 85 down-regulated, were identified. The lincRNA accounted for the most proportion of differentially expressed lncRNAs, which was in accordance with previous study (23). To further validate the expression level of identified exosomal lncRNA, six of them, one up-regulated and five down-regulated, were randomly selected to perform qRT-PCR analysis. However, no significant difference was detected between T1DM patients and control subjects. The relatively limited sample size may contribute to the negative results more or less. Nevertheless, there were still 156 differentially expressed lncRNAs to be further test in future studies. We believe that some of these may have the potential to become novel biomarkers for T1DM given the fact that both exosome (22) and lncRNA (15) have been reported to be significantly associated with T1DM. It has been indicated that islet-derived exosomes can activate immune response and lead to autoimmune attack (24). Also, T lymphocyte-derived exosomes are associated with beta-cell dysfunction and death (25). Some studies also evaluated the diagnostic potential of exosomes in the context of T1DM. It has been demonstrated that human islet-derived exosomal RNAs were differentially expressed when exposed to proinflammatory cytokines (26). Another study investigated the plasma-derived exosomes and reported a distinct exosomes miRNAs signature in patients with long-duration T1DM (27). Some studies have also investigated the potential role of lncRNA in the T1DM pathogenesis. LncRNAs usually expressed in a cell-specific manner. Previous transcriptome profiling studies have identified more than 1000 islet-specific lncRNAs (28, 29). The dysfunction of lncRNAs may lead to autoimmune response and alter the progression of T1DM (30, 31). A recent whole genome RNA sequencing study in circulating leukocytes identified 393 differentially expressed lncRNAs, potentially providing novel targets for diagnosis and treatment of T1DM (32). Another study conducted a comparative analysis of the expression profiles of lncRNA by analyzing published microarray data set and proposed a specific 26-lncRNA signature, which could be used to effectively identify T1DM susceptible individuals (33). In addition, *in vitro* experiment using MIN6 cells found that the expression of lncRNA was modified by cytokine treatment and overexpression of these lncRNA favored beta-cell apoptosis, indicating dysregulation of lncRNA contributes to the beta-cell failure during the development of T1DM (18). Furthermore, some specific lncRNAs have been reported to be implicated in beta-cell function and T1DM. It has been indicated that lncRNA MALAT1 could induce beta-cell dysfunction through inhibiting the expression of PDX-1 by reducing its H3 histone acetylation (34). The lncRNA Lnc13 could regulate the inflammation of pancreatic beta-cell by allele-specific stabilization of STAT1 mRNA (35). All the aforementioned studies suggested that both exosome and lncRNA were involved in the T1DM pathogenesis.

Next, to predict the potential biological functions of identified differentially expressed lncRNAs, we performed GO enrichment analysis and the KEGG pathway analysis. The terms such as activation of phospholipase D activity, neuronal cell body membrane, calcium sensitive guanylate cyclase activator activity, and metal ion binding were significantly enriched in the identified differentially exosomal lncRNAs. These terms were associated with diabetes or its complication to some extent. For instance, it has been shown that glycosylphosphatidylinositol-specific phospholipase D level were correlated to the insulin resistance of adipose tissue in obese subjects (36). Also, the expression of glycoprotein phospholipase D (GPLD1) was found to be increased in mouse models of T1DM (37, 38), and GPLD1 levels in autoimmune diabetes were higher than that in T2DM or healthy controls (39).

KEGG pathway analysis indicated that the differentially expressed lncRNAs were involved in oxidative phosphorylation, Parkinson’s disease (PD) and pathways in cancer. PD is also a multifactorial disease and both genetic and environmental factors contribute to the pathogenesis of this progressive neurodegenerative disease (40). The primary pathogenic mechanisms of PD remain unclear. However, some pathophysiological processes, such as mitochondrial dysfunction, oxidative stress, and chronic inflammation, are identified to be associated with PD (41). These processes are also involved in the onset and development of T1DM (42, 43), which suggested that PD and T1DM might share some common pathological features. Interestingly, another progressive neurodegeneration disease, Alzheimer’s disease (AD), has been proposed as “type 3 diabetes mellitus” because of the shared molecular and cellular characteristics with T1DM and T2DM (44).

LncRNAs were frequently precursor RNAs of miRNAs. We identified the precursor lncRNAs of miRNA through comparing miRNA sequence in miRbase database. Some identified miRNAs such as miR-21 (45) and miR-424 (46) has been implicated in the progression of T1DM. Therefore, the lncRNA combined miRNA might contribute to the T1DM collectively.

Both exosome and lncRNA have been implicated in the progression of T1DM. However, the exact role of exosomal lncRNA in T1DM lacks full investigation and research. The present study identified the expression profiles of exosomal lncRNA and laid foundation for further study in this field. There are some limitations of our study. First, the sample size was relatively small. Future study should include more samples to decrease the random errors. Second, we only selected six lncRNAs to validate their expression level by qRT-PCR and the sample size was relatively small. Further study should focus on more lncRNAs based on the sequencing results and assess the biomarker potential of exosomal lncRNAs in a larger sample set. Third, we didn’t investigate the function of exosomal lncRNAs and some *in vitro* or *in vivo* study are required in future study.

In conclusion, this study identified the characteristics of the plasma-derived exosomal lncRNA transcriptome of T1DM for the first time and threw insights into the biomarker potential and pathogenic factor of exosomal lncRNA in T1DM.

## Data Availability Statement

The original contributions presented in the study are publicly available. This data can be found here: [https://db.cngb.org/cnsa/ accession number CNP0002574].

## Ethics Statement

The studies involving human participants were reviewed and approved by the Ethics Committee of the Second Xiangya Hospital. The patients/participants provided their written informed consent to participate in this study.

## Author Contributions

ZZ, ZX, and HP conceived and designed the experiments. HP, WF, JLi, YW, SL, JLin, GH, and XL collected samples. HP, XS, and WF performed the experiments and analyzed the data. HP wrote the manuscript. All authors contributed to the article and approved the submitted version.

## Funding

This work was supported by the National Natural Science Foundation of China (grant numbers 82070813, 81873634, 81400783), the National Key R&D Program of China (grant numbers 2016YFC1305000, 2016YFC1305001, 2018YFC1315603), and the Hunan Province Natural Science Foundation of China (grant numbers 2018JJ2573, 2020JJ2053).

## Conflict of Interest

The authors declare that the research was conducted in the absence of any commercial or financial relationships that could be construed as a potential conflict of interest.

## Publisher’s Note

All claims expressed in this article are solely those of the authors and do not necessarily represent those of their affiliated organizations, or those of the publisher, the editors and the reviewers. Any product that may be evaluated in this article, or claim that may be made by its manufacturer, is not guaranteed or endorsed by the publisher.
